# Web-based Investigation of Multistate Salmonellosis Outbreak

**DOI:** 10.3201/eid1104.040997

**Published:** 2005-04

**Authors:** Padmini Srikantiah, Dean Bodager, Bill Toth, Taha Kass-Hout, Roberta Hammond, Sara Stenzel, R.M. Hoekstra, Jennifer Adams, Susan Van Duyne, Paul S. Mead

**Affiliations:** * Centers for Disease Control and Prevention, Atlanta, Georgia, USA;; † Florida Department of Health, Tallahassee, Florida, USA;; ‡ Minnesota Department of Health, Minneapolis, Minnesota, USA

**Keywords:** Salmonella, Web-based, immunocompromised, transplant, tomatoes, dispatch

## Abstract

We investigated a large outbreak of *Salmonella enterica* serotype Javiana among attendees of the 2002 U.S. Transplant Games, including 1,500 organ transplant recipients. Web-based survey methods identified pre-diced tomatoes as the source of this outbreak, which highlights the utility of such investigative tools to cope with the changing epidemiology of foodborne diseases.

The epidemiology of foodborne illnesses is influenced by a variety of factors, some of which have changed dramatically in recent years. The increased availability of preprocessed foods and the improved survival of persons with immune defects have affected the sources and nature of foodborne illness (*1**–**4*). Increased mobility of Americans through interstate travel has complicated the identification and investigation of outbreaks. New technologies for outbreak investigation have the potential to greatly assist public health officials in successfully managing these changing factors. We describe an outbreak of *Salmonella enterica* serotype Javiana infections affecting a large group of geographically dispersed organ transplant recipients. The prompt and successful investigation of this outbreak was facilitated by the use of Web-based surveys.

## The Study

On July 16, 2002, the Minnesota Department of Health identified 2 cases of *S*. Javiana infection among persons who attended the 2002 U.S. Transplant Games, an Olympics-style athletic competition among recipients of solid organ and bone marrow transplants that was held June 25–29 at theme park A in Orlando, Florida. Isolates from the 2 patients were indistinguishable when subtyped by pulsed-field gel electrophoresis (PFGE). Approximately 6,000 people, including 1,500 transplant recipient athletes, attended the 2002 Transplant Games.

To identify additional cases, state health departments were asked to report any *S*. Javiana isolates with a PFGE pattern indistinguishable from the outbreak strain. To develop hypotheses about potential sources of infection, we conducted in-depth telephone interviews with several persons who were identified with culture-confirmed illness.

On the basis of interview results, we conducted a Web-based cohort study among Transplant Games attendees to identify risk factors for infection by using eQuest, a software package developed by the Centers for Disease Control and Prevention (CDC) that allows rapid development of Web-based surveys (*5*). Using email addresses provided to us by the Transplant Games organizers, we electronically distributed a message on July 20 to attendees (including athletes and spectators), requesting that they complete an outbreak survey. We included information about salmonellosis and its treatment and provided a link in the email to the secure Web site containing the outbreak survey. In each survey respondent's household, we collected information for a single person visiting Orlando, regardless of whether he or she had been ill. A case was defined as fever or diarrhea with onset between June 25 and July 7 in a person who visited Orlando. Submitted answers were automatically stored in a secure electronic database and linked only to a random survey number.

To identify the specific food item responsible for illness, we performed a Web-based case-control study. On July 31, we distributed a survey containing detailed questions about specific food items available in theme park A to persons who had responded to the first survey. Case-patients were questioned about food items eaten in the 3 days before illness onset. Controls were defined as well survey respondents, and all were questioned about the middle 3 days of the Transplant Games (June 26–28).

Plant X, the processing plant that supplied tomatoes to theme park A, was inspected on August 13. Molecular subtyping of confirmed *S*. Javiana isolates was performed at state public health laboratories (*6*). Diced Roma tomatoes from unopened boxes that had been stored frozen at theme park A were cultured at the Florida State Public Health Laboratory.

Statistical analyses were conducted by using SAS software version 8.2 (SAS Institute Inc., Cary, NC, USA) to calculate odds ratios (OR) and 95% confidence intervals (CI). Multivariable logistic regression analyses were conducted for variables that were significantly associated with illness.

Through laboratory surveillance, 21 additional *S*. Javiana infections with indistinguishable PFGE patterns were identified in 10 states, for a total of 23 identified culture-confirmed cases. Dates of illness onset were from June 24 to July 8. Of 22 patients for whom travel information was collected, 19 reported visiting theme park A in the last week of June; 16 visited theme park A but did not report any contact with the Transplant Games, which suggests a true outbreak of *S*. Javiana infections among visitors to the theme park.

An electronic link to the Web-based cohort study survey was distributed on July 20, 2002, to 1,100 Transplant Games attendees. Among these 1,100, we received survey responses from 369 persons (34%) in 42 states; 80% responded within 48 hours. Of the 369, a total of 82 (22%) reported illness and 41 (53%) were female. The median age of ill respondents was 47 years (range 4–71 years); 48 (59%) were transplant recipients. Dates of symptom onset were June 26–July 7 (Figure). Predominant symptoms included diarrhea (93%), abdominal pain (79%), and fever (51%). Three respondents (4%) had been hospitalized. No deaths were reported to the organizers of the Transplant Games or CDC. Among ill respondents, 75 (91%) reported eating food items at specific food courts in theme park A.

**Figure:**
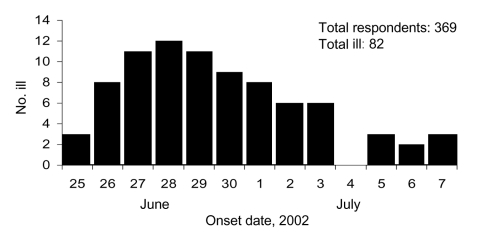
Diarrheal illness among attendees of the 2002 U.S. Transplant Games in Orlando, Florida.

The Web-based case-control study was distributed on July 31 to the 369 persons who responded to the first survey. By August 2, a total of 222 persons (60%) responded. Of 217 valid responses, 41(19%) were ill persons who met the case definition; the remaining 176 were healthy controls. Ill persons were significantly more likely to report eating dishes containing diced Roma tomatoes than were well persons (44% of ill vs. 15% of well, OR = 4.3, 95% CI 2.1–9.1). Other food items that were significantly associated with illness on univariate analysis were dishes containing shredded iceberg lettuce (OR = 3.7, 95% CI 1.8–7.4), pre-shredded cheddar cheese (OR = 2.9, 95% CI 1.5–5.9), fresh ground beef (OR = 3.0, 95% CI 1.4–6.4), and pre-sliced beefsteak tomatoes (OR = 4.6, 95% CI 1.1–19.4) (Table). In multivariable logistic regression modeling, only diced Roma tomatoes remained independently associated with illness at the 0.05 significance level.

**Table:** Univariate analysis of food exposures among ill and well persons who attended the Transplant Games in Orlando, Florida*

Exposure	Ill (N = 41), n (%)	Well (N = 176), n (%)	OR	95% CI	p value
Shredded iceberg lettuce	24 (59)	49 (28)	3.7	1.8–7.4	0.0002
Shredded cheddar cheese	22 (54)	50 (28)	2.9	1.5–59	0.002
Diced Roma tomatoes	18 (44)	27 (15)	4.3	2.1–9.1	<0.0001
Fresh ground beef	14 (34)	26 (15)	3.0	1.4–6.4	0.004
Pre-sliced beefsteak tomatoes	4 (10)	4 (2)	4.6	1.1–19.4	0.02
Frozen ground beef	12 (29)	31 (18)	1.9	0.9–4.2	0.09

*OR, odds ratio; CI, confidence interval.

Diced Roma tomatoes were supplied to theme park A from plant X, where whole Roma tomatoes were mechanically diced and washed in a manually chlorinated recycled water tank. Levels of chlorine in the tank were variable (≈1.5–3.5 ppm free chlorine), providing potential opportunity for the amplification of any existent microbial contamination. Review of invoices showed that diced Roma tomatoes used in food courts patronized by persons with outbreak-related illness were processed at plant X from June 20 through July 3. No diced tomatoes from the implicated lots were available for testing. Microbiologic evaluation of an unopened box of plant X diced Roma tomatoes processed on July 12 indicated the presence of fecal coliforms (150–1,000 CFU/g).

## Conclusions

The nature of this outbreak highlights several changing features of foodborne disease epidemiology, including the enhanced mobility of persons through air travel, an increasing reliance on pre-processed foods, and an expanding immunocompromised population at risk. Through a Web-based investigation, we were able to rapidly identify the source of this outbreak and inform an immunocompromised population of its potential risk for illness. Our approach allowed us to contact and question several hundred geographically dispersed persons in a matter of days. Survey respondents' answers were automatically stored in a secure electronic database, eliminating the need for data entry. With the development of questionnaire templates, a public health official could select sets of questions and pre-coded answers from pull-down menus and modify them to design an outbreak-specific, Web-based questionnaire that is automatically linked to an electronic database (*5*). These methods make it increasingly possible to design and post a Web-based investigative tool for wide distribution within hours.

Our investigation has several limitations. Use of a Web-based investigation tool limited responses to only those Transplant Games attendees with known email addresses and Internet access. The initial response rate to our survey was only 34%; households with ill persons may have been more likely to respond to our Web-based survey. However, most (>75%) respondents to both surveys were from households in which no one had experienced illness, which provided us with a sufficient number of responses from both well and ill persons to identify the source of the outbreak. Hospitalized and severely ill persons may have been too sick to respond to the survey or may have been unable to access the Internet, which limited our ability to calculate accurate hospitalization or attack rates among persons attending the Transplant Games. Although isolation of *S*. Javiana from plant X diced tomatoes would have strengthened our findings, the tomatoes tested were processed well after the outbreak period, when levels of contamination may have differed considerably.

As use of the Internet becomes more widespread for participation in regional, national, and international conferences, groups, and listservs, electronic mail cohorts are becoming more commonplace. The development of Web-based public health investigative tools can facilitate future investigations of outbreaks affecting geographically dispersed persons who may be part of an electronic mail cohort. A Web-based approach to data collection can also play a critical role in rapidly sharing data in outbreaks involving multiple jurisdictions (*7*). The growing use of Web-based technologies in public health investigations will have to be balanced with the need to protect the privacy of personal information in the online environment (*8**,**9*). The careful application of emerging technologies and conventional epidemiologic techniques can help public health officials effectively cope with the multitude of changing factors that shape public health in the United States.
